# Rapid recurrence of squamous cell carcinoma at a lymphocele after nephroureterectomy: A rare case report

**DOI:** 10.1002/iju5.12259

**Published:** 2021-02-15

**Authors:** Kosuke Ogawa, Yousuke Shimizu, Shoko Uketa, Noriaki Utsunomiya, Satsuki Asai, Misa Ishihara, Kimio Hashimoto, Sojun Kanamaru

**Affiliations:** ^1^ Department of Urology Kobe City Nishi‐Kobe Medical Center Kobe Hyogo Japan; ^2^ Department of Pathology Kobe City Nishi‐Kobe Medical Center Kobe Hyogo Japan

**Keywords:** lymphocele, metastasis, squamous differentiation, ureteral cancer, urothelial carcinoma

## Abstract

**Introduction:**

Lymphoceles are sometimes formed after pelvic lymph node dissection. However, recurrence at lymphoceles has not been reported previously. Here, we report a case of rapid prognosis of the recurrence at a lymphocele after nephroureterectomy.

**Case presentation:**

A 78‐year‐old man underwent retroperitoneoscopic radical nephroureterectomy with pelvic lymphadenectomy for left ureteral urothelial carcinoma. The histopathological diagnosis was high‐grade invasive urothelial carcinoma with squamous differentiation. Follow‐up computed tomography at 3 months postoperatively showed a lymphocele with a small solid component, in the left pelvic region. At 7 months postoperatively, he presented with severe fatigue, and computed tomography showed a solid tumor had replaced the lymphocele. Computed tomography‐guided biopsy was performed and histopathological diagnosis was squamous cell carcinoma.

**Conclusion:**

This report provides support for possible recurrence at the lymphocele after nephroureterectomy. If lymphocele occurs after surgery for malignant disease, it is recommended to follow up with the possibility of recurrence in the lymphatic cysts in mind.

**Abbreviations & Acronyms**
CT: computed tomography
MRI: magnetic resonance imaging
SCC: squamous cell carcinoma
UVJ: ureterovesical junction

**Keynote message**

Lymphoceles are often detected by follow‐up CT after surgery for malignant disease. They often present some symptoms, but they are usually not related to cancer. Our report suggests if lymphocele with a solid component occurs after surgery for malignant disease, it is recommended to follow up with the possibility of recurrence in the lymphatic cysts in mind.

## Introduction

Carcinoma of the upper urinary tract is an uncommon urothelial malignancy.^1^ Ureteral cancer accounts for only 5–10% of urothelial cancers.^2^, ^3^ Squamous differentiation is the most common histological variant of urothelial cancer, and occurs in up to 20% of cases of urothelial carcinoma.^4^, ^5^ It is known to be an aggressive histological type.^5^, ^6^, ^7^ A lymphocele is a lymphatic fluid collection arising as a consequence of surgical dissection and inadequate closure of lymphatic vessels. In most cases, pelvic lymphoceles are asymptomatic but sometimes they show symptoms of infection, fever, and pain.^8^ Although, these symptoms have been reported, there are no reports of metastasis at lymphoceles. Here, we report what is believed to be the first case of rapid metastasis of urothelial carcinoma at the lymphocele after nephroureterectomy.

## Case presentation

**Fig. 1:**
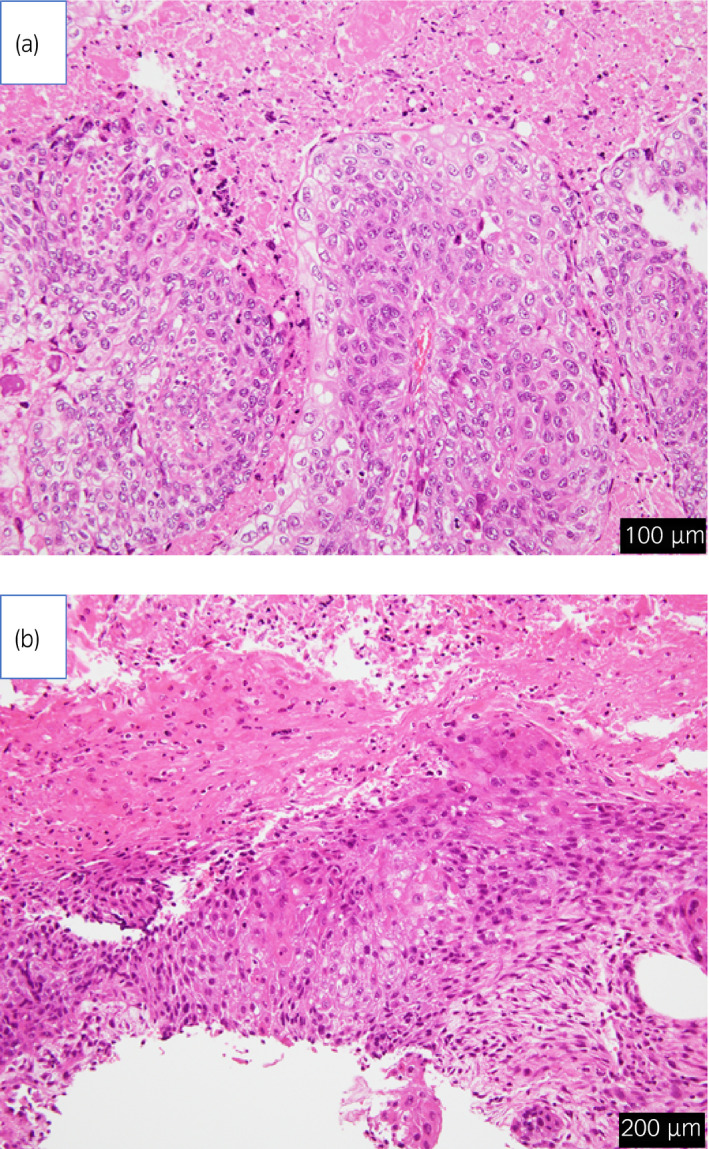
(a) There are invasive urothelial carcinoma, mainly composed of well‐differentiated SCC (hematoxylin‐eosin, bar = 100 μm). (b) There are well‐differentiated SCC and the histopathological findings are similar to those of ureteral cancer (a) (hematoxylin‐eosin, bar = 200 μm).

**Fig. 2:**
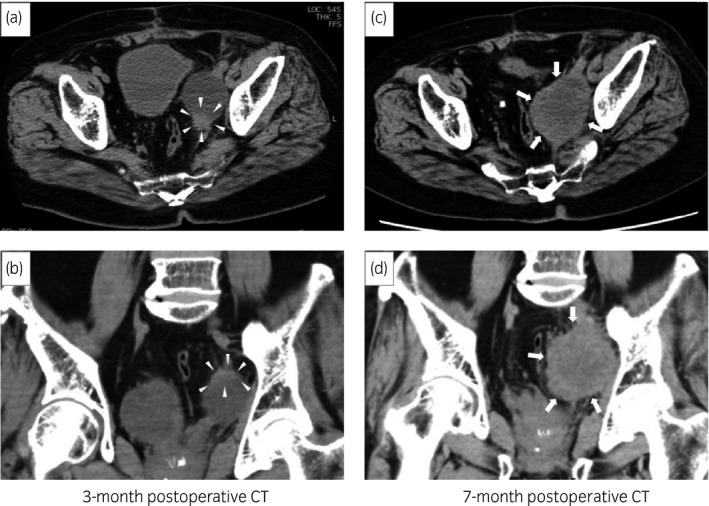
The postoperative 3‐month CT scan (a: axial view, b: coronal view) showed a lymphocele with a small solid component in the wall, in the left pelvic region. The coronal view showed the solid component area was distant from the UVJ. The postoperative 7‐month CT scan (c: axial view, d: coronal view) showed a solid mass had replaced the lymphocele in the left pelvic region.

**Fig. 3:**
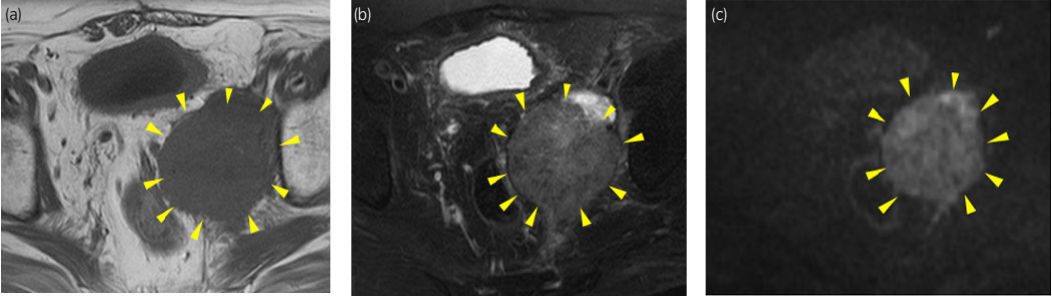
MRI showed that the mass with arrow pointing was isointense on (a) T1‐ and (b) T2‐weighted imaging and hyperintense on (c) diffusion‐weighted imaging.

A 75‐year‐old man underwent resection for lung cancer. At 3 years after surgery, follow‐up enhanced CT revealed a mass with strong enhancement from just above the UVJ extending 30 mm length in the left ureter. We performed ureteroscopic biopsy and the histopathological diagnosis was urothelial carcinoma. No metastatic lesions were found on whole‐body CT scan. He was diagnosed with left ureteral cancer (cT2N0M0). He underwent retroperitoneoscopic radical nephroureterectomy with bladder cuff excision and lymphadenectomy of the ipsilateral common iliac, external iliac, internal iliac, and obturator lymph nodes. The tumor was 43 mm in diameter, and the histopathological diagnosis was invasive urothelial carcinoma, mainly composed of well‐differentiated SCC (pT3N0) (Fig. 1a). The resection margin was negative and lymph node metastasis was not detected. At 3 months postoperatively, follow‐up CT showed a lymphocele (43 × 60 × 67 mm) with a small solid component in the wall, in the left pelvic region (Fig. 2a). The solid component area was distant from the primary lesion located in the UVJ (Fig. 2b). At that time, the solid component was considered to be only lymphocele wall thickening. At 6 months postoperatively, the patient underwent transurethral resection for recurrent bladder cancer. The histopathological diagnosis was invasive urothelial carcinoma (pT1). At 7 months postoperatively, he was admitted to our hospital because of sever fatigue. CT showed that a solid mass had replaced the lymphocele in the left pelvic region (Fig. 2c,d). We suspected an infectious cyst or tumor metastasis, and MRI was performed for further analysis. MRI showed that the mass was isointense on T1‐ and T2‐weighted imaging and hyperintense on diffusion‐weighted imaging (Fig. 3). CT‐guided biopsy was performed and histopathological diagnosis was well‐differentiated SCC (Fig. 1b). The histopathological findings were similar to those of ureteral cancer; therefore, we diagnosed the pelvic mass as ureteral cancer metastasis. Unfortunately, his general condition deteriorated, so he chose not to receive any additional therapy for the metastatic tumor.

## Discussion and conclusions

The incidence rate of lymphocele formation after nephroureterectomy is not reported in the literature. In a clinical setting, we often detect lymphoceles by postoperative follow‐up CT. Lymphocele formation after pelvic lymph node dissection is the common complication. Symptomatic lymphoceles are identified in 0–8% of patients and the development of asymptomatic lymphoceles has been reported in up to 1.5–51% of patients.^8^, ^9^, ^10^, ^11^, ^12^, ^13^ The presenting symptoms were fever, abdominal pain, genital swelling, groin pain, abdominal swelling, leg edema, and deep vein thrombosis.^8^, ^9^, ^13^ In our case, the lymphocele was formed in the retroperitoneal cavity after nephroureterectomy and the patient had severe fatigue as the recurrence at the lymphocele grows. Upper tract urothelial carcinoma commonly recur at the urinary tract, lymph node, liver, bone, and lung recurrences, and the lymph node metastasis was observed in 27% of patients who underwent nephroureterectomy.^14^ As for the isolated local recurrences, it observed in 3% of patients.^15^ Especially, squamous differentiation in upper urinary tract urothelial carcinoma presents at a higher clinical stage and appears to represent more aggressive disease than do other histological types.^7^, ^16^ The nodal metastatic rate is significantly higher than pure urothelial carcinoma.^6^

There are no reports of cancer recurrence at lymphoceles associated with lymph node dissection.^12^ CT showed a small solid component at the lymphocele wall at 3 months postoperatively, and the lymphocele was replaced by a solid mass at 7 months postoperatively, that did not appear to compress the lymphocele wall. There are hypotheses as to the mechanism of this metastasis. It is dissemination, lymphatic metastasis, or cyst wall metastasis. The histopathological examination at nephroureterectomy showed that the resection margins were negative and no lymph node metastasized. And the solid component area was distant from the primary lesion located in the UVJ on 3 months postoperative CT. These findings suggest the possibility of cancer recurrence at the lymphocele, not local recurrence. Unfortunately, the exact site and mechanism of recurrence could not be proved because the recurrent tumor including the lymphocele was not resected. It was diagnosed from a biopsy. At 3 months postoperatively, our radiologist had diagnosed as a lymphatic cyst and the wall thickening was not considered a significant finding. However, we should have been considered other radiographic modalities including MRI or positron emission tomography‐CT when a small solid component was observed at the lymphocele wall.

In conclusion, this is a rare case report suggesting the possibility of recurrence at lymphocele after nephroureterectomy. Lymphoceles have the potential to become metastatic sites. If lymphocele with a solid component occurs after surgery for malignant disease, it is recommended to follow up with the possibility of recurrence in the lymphatic cysts in mind.

## Conflict of interest

The authors declare no conflict of interest.

## Ethics

We obtained written informed consent from the patient.

## References

[1] Roupret M , Babjuk M , Comperat E *et al*. European guidelines on upper tract urothelial carcinomas: 2013 update. Eur. Urol. 2013; 63: 1059–71. 23540953 10.1016/j.eururo.2013.03.032

[2] Munoz JJ , Ellison LM . Upper tract urothelial neoplasms: incidence and survival during the last 2 decades. J. Urol. 2000; 164: 1523–5. 11025695

[3] Joung JY , Lim J , Oh CM *et al*. Current trends in the incidence and survival rate of urological cancers in Korea. Cancer Res. Treat. 2017; 49: 607–15. 27658388 10.4143/crt.2016.139 PMC5512381

[4] Rink M , Robinson BD , Green DA *et al*. Impact of histological variants on clinical outcomes of patients with upper urinary tract urothelial carcinoma. J. Urol. 2012; 188: 398–404. 22698626 10.1016/j.juro.2012.04.009

[5] Liu Y , Bui MM , Xu B . Urothelial carcinoma with squamous differentiation is associated with high tumor stage and pelvic lymph‐node metastasis. Cancer Control 2017; 24: 78–82. 28178718 10.1177/107327481702400113

[6] Makise N , Morikawa T , Kawai T *et al*. Squamous differentiation and prognosis in upper urinary tract urothelial carcinoma. Int. J. Clin. Exp. Pathol. 2015; 8: 7203–9. 26261615 PMC4525949

[7] Berz D , Rizack T , Weitzen S , Mega A , Renzulli J , Colvin G . Survival of patients with squamous cell malignancies of the upper urinary tract. Clin. Med. Insights Oncol. 2012; 6: CMO.S8103. 10.4137/CMO.S8103 PMC3256977 22253551

[8] Keskin MS , Argun OB , Obek C *et al*. The incidence and sequela of lymphocele formation after robot‐assisted extended pelvic lymph node dissection. BJU Int. 2016; 118: 127–31. 26800257 10.1111/bju.13425

[9] Ploussard G , Briganti A , de la Taille A *et al*. Pelvic lymph node dissection during robot‐assisted radical prostatectomy: efficacy, limitations, and complications – a systematic review of the literature. Eur. Urol. 2014; 65: 7–16. 23582879 10.1016/j.eururo.2013.03.057

[10] Horovitz D , Lu X , Feng C , Messing EM , Joseph JV . Rate of symptomatic lymphocele formation after extraperitoneal vs transperitoneal robot‐assisted radical prostatectomy and bilateral pelvic lymphadenectomy. J. Endourol. 2017; 31: 1037–43. 28741376 10.1089/end.2017.0153

[11] Orvieto MA , Coelho RF , Chauhan S , Palmer KJ , Rocco B , Patel VR . Incidence of lymphoceles after robot‐assisted pelvic lymph node dissection. BJU Int. 2011; 108: 1185–90. 21489117 10.1111/j.1464-410X.2011.10094.x

[12] Chen H‐H , Ting W‐H , Lin H‐H , Hsiao S‐M . Predictors of lymphoceles in women who underwent laparotomic retroperitoneal lymph node dissection for early gynecologic cancer: a retrospective cohort study. Int. J. Environ. Res. Public Health 2019; 16: 936. 10.3390/ijerph16060936 PMC6466375 30875912

[13] Gotto GT , Yunis LH , Guillonneau B *et al*. Predictors of symptomatic lymphocele after radical prostatectomy and bilateral pelvic lymph node dissection. Int. J. Urol. 2011; 18: 291–6. 21306438 10.1111/j.1442-2042.2010.02710.x

[14] Mao Y , Kilcoyne A , Hedgire S *et al*. Patterns of recurrence in upper tract transitional cell carcinoma: imaging surveillance. AJR Am. J. Roentgenol. 2016; 207: 789–96. 27382922 10.2214/AJR.16.16064

[15] Margulis V , Shariat SF , Matin SF *et al*. Outcomes of radical nephroureterectomy: a series from the Upper Tract Urothelial Carcinoma Collaboration. Cancer 2009; 115: 1224–33. 19156917 10.1002/cncr.24135

[16] Lee YJ , Moon KC , Jeong CW , Kwak C , Kim HH , Ku JH . Impact of squamous and glandular differentiation on oncologic outcomes in upper and lower tract urothelial carcinoma. PLoS One 2014; 9: e107027. 25191845 10.1371/journal.pone.0107027 PMC4156382

